# Validation of the Persian Translation of the Swallowing Disturbance Questionnaire in Parkinson's Disease Patients

**DOI:** 10.1155/2014/159476

**Published:** 2014-10-27

**Authors:** Ali Rajaei, S. Abolfazl Azargoon, Mohammad Hussein Nilforoush, Ebrahim Barzegar Bafrooei, Fereshteh Ashtari, Ahmad Chitsaz

**Affiliations:** ^1^Isfahan Neuroscience Research Center, Isfahan University of Medical Sciences, Isfahan 8174673461, Iran; ^2^Mahoor Clinic of Speech and Language Pathology, Iran; ^3^Audiology Department, School of Rehabilitation Sciences, Isfahan University of Medical Sciences, Isfahan 8174673461, Iran; ^4^Department of Speech and Language Pathologist, Tehran University of Medical Sciences, Tehran 141556559, Iran; ^5^Department of Neurology, Isfahan University of Medical Sciences, Isfahan 8174673461, Iran

## Abstract

Dysphagia, as a common finding in Parkinson's disease (PD) patients, was estimated to be present in 80–95% of this population during different stages of the disease. The Swallowing Disturbance Questionnaire (SDQ) was created as a self-rated dysphagia screening tool in PD. According to the guidelines for cross-cultural adaptation, Persian version of this questionnaire (SDQ-P) was developed. 59 Persian patients (39 men and 20 women) participated in the study. They responded to the SDQ-P and underwent videofluoroscopic swallowing study (VFSS). Aspiration during VFSS was compared with questionnaire results for each individual. Cronbach's alpha coefficient for the questionnaire was 0.86 and based on SDQ-P 15 patients (25.4%) were dysphagic, while 10 patients (16.9%) showed aspiration during VFSS. SDQ-P sensitivity and specificity in predicting aspiration were 96.7 and 91.2%; therefore, the SDQ-P could be a prognostic tool for aspiration. The positive predictive value (PPV), the negative predictive value (NPV), and the pre- and posttest probabilities of aspiration were 0.67, 1, 16.9%, and 66.7%, respectively. In summary, this study demonstrated the reliability and also the feasibility of SDQ-P for screening of aspiration in Iranian patients with PD. Further evaluation of SDQ-P in larger subject population would be suggested.

## 1. Introduction

Parkinson's disease (PD), resulting from progressive degeneration of brainstem, midbrain, subcortical, and cortical neurons [1], can cause oropharyngeal dysphagia, as a common finding, in up to 80% of patients during the first stages and up to 95% in advanced stages of the disease [2, 3]. Quality of life in PD patients is also thought to be significantly affected by dysphagia [4]. Fiberoptic endoscopic evaluation of swallowing (FEES), videofluoroscopic swallowing study (VFSS), and diagnostic ultrasound are considered as the gold standards for the diagnosis of dysphagia [5–7], but exposure to X-rays, insufficient specialist, and expensive charges are among the disadvantages of these techniques. When VFSS or other instrumental examinations are not the first choice, a standardized dysphagia rating questionnaire could be practical [8]. The SWAL-QOL [9], M. D. Anderson Dysphagia Inventory (MDADI) [10], Dysphagia in Multiple Sclerosis (DYMUS Questionnaire) [11], the Dysphagia Handicap Index [12], the Deglutition Handicap Index [13], and the Swallowing Disturbance Questionnaire (SDQ) [14] have been introduced until now. SDQ, comprising 15 items, was presented as a self-rated dysphagia screening tool in PD patients. It was also investigated on patients with dysphagia associated with various other etiologies [15] and was previously translated to Japanese [16]. In order to be appropriate for widespread use, further extensive testing of this questionnaire in other languages was advised [17]. Thus, Persian version of SDQ (SDQ-P) was created and its reliability in relation to the aspiration status of patients in accordance with Yamamoto et al. study [16] during videofluoroscopic swallowing study (VFSS) was examined.

## 2. Subjects and Methods

### 2.1. Subjects

59 Iranian patients (mean age, 66.0 ± 9.7 years; mean disease duration 5.1 ± 3.9 years; 39 men, 20 women) were selected among 76 patients who were referred to our neurology center either for evaluating or treatment of Parkinsonism between February 10, 2013, and October 28, 2013, participated in our study.

All data samples had a Mini-Mental State Examination (MMSE) [18] score above 27. None of them received swallowing therapy before our study and five patients had a history of aspiration pneumonia. The inclusion criteria were (1) being diagnosed with clinically definite PD [19], (2) being able to fill out the questionnaire by him/herself; and we excluded: (1) PD patients who suffer from other neurodegenerative disorders, found by different imaging techniques, (2) patients or with history of other diseases that could potentially cause dysphagia, and (3) those with feeding tubes. All the study phases were performed during patient's “on” state. The study was approved by ethical committee of our center, and all the patients gave their written informed consent before beginning the study in accordance with the Declaration of Helsinki.

### 2.2. Persian Version of SDQ

The guidelines for the cross-cultural adaptation of self-report measures were considered for creating SDQ-P [20]. Having the permission of the original author, two translators translated the questionnaire into Persian and then a native English language speaker reverse-translated it into English. The back translation was sent to the original author for proofreading. Finally, we were granted to use the complete SDQ-P. All items of SDQ-P were uniformly in English version and all the patients answered the SDQ-P before VFSS.

### 2.3. Videofluoroscopic Swallowing Study

VFSS was performed at lateral plane. Patients were seated upright just as the posture during their meals. Four bolus types were administered including 5 mL of thin liquid from a spoon, thin liquid by a cup which was self-administered, a semisolid (5 mL) from a spoon (puree), and finally half a cookie [21]. Patient's videos were recorded on DVD at 30 frames/s. After the test, patient's records (except the first swallow from the patient) were evaluated for detecting aspiration by an assessor using Penetration-Aspiration Scale (PAS) (Table 1) [22]. Thirty-seven of VF records were reevaluated by the same assessor and the same 37 videos were also evaluated by another assessor in order to measure the reliability of VF records evaluation.

### 2.4. Data Analysis

Hoehn-Yahr (H&Y) stage [23], age, and sex of patients with and without aspiration were compared using Mann-Whitney *U* test. *κ* coefficient was used for testing the interrater and intrarater reliability of VF records evaluation. The cutoff point for total score of the questionnaire was determined by the receiver operating characteristic (ROC) analysis. Finally, patient's aspiration statuses during VFSS and SDQ-P results were compared using Fisher's exact test. All analyses were performed by IBM SPSS (ver. 18.0).

## 3. Results

All the items were completely clear for our patients and there were no questions about content of SDQ-P. 10 patients (16.9%) showed aspiration during VFSS (Table 2). There were no significant differences between patients with or without aspiration in terms of age, gender, and H&Y stage (*P* = 0.347 for age, *P* = 0.657 for gender, and *P* = 0.079 for H&Y stage).

Internal consistency (*κ* coefficient 1.00) and consistency between assessors (*κ* coefficient 0.93, 95% confidence interval (CI) 0.89–0.95) were significant. Thus, the evaluation of aspiration was highly consistent. Cronbach's alpha coefficient for the 15 questions of the questionnaire was 0.86 that confirmed the reliability of SDQ-P.

**Figure 1:**
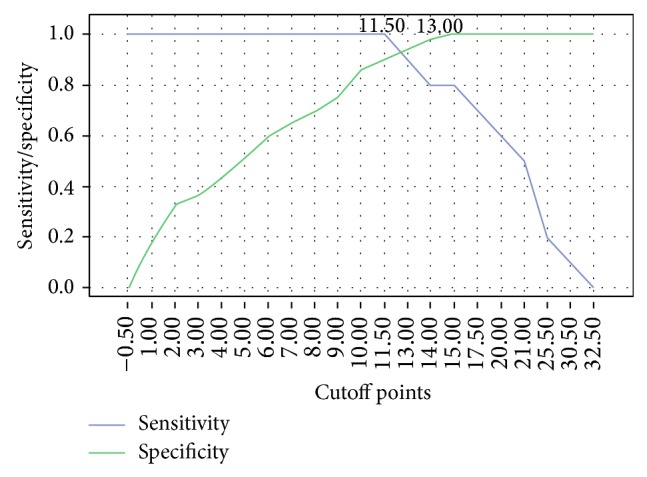
The receiver operating characteristics curve. The SDQ-P cutoff score is equal to 12.59 points, the specificity is 91.2%, and the sensitivity is 96.7% (*x*-axis: the cutoff points for the SDQ-P and *y*-axis: the sensitivity and the specificity).

In ROC analysis, the sensitivity and specificity curves crossed at 12.59 (Figure 1). We determined 12 points as an appropriate cutoff point for SDQ-P because nearby 12.59 points the sensitivity was descending while the specificity was ascending. Then, 15 patients (25.4%) with the scores more than 12 points were assessed to have dysphagia. SDQ-P sensitivity and specificity in predicting aspiration were 96.7 and 91.2%; therefore, the SDQ-P could be a prognostic tool for aspiration. The positive predictive value (PPV), the negative predictive value (NPV), and the pre- and posttest probabilities of aspiration were 0.67, 1, 16.9%, and 66.7%, respectively (Table 3).

## 4. Discussion

Prolonged oral-pharyngeal transit time and abnormal lingual control [2], longer esophageal transit time [24], sialorrhea [25], impaired laryngeal excursion [2, 26], and malfunction of esophageal sphincters [27] are known as the common swallowing disturbances in patients with Parkinson's disease. Disturbed quality of life [4] and high risk of aspiration pneumonia, as the frequent cause of death in PD [28, 29], are devastating consequences of the dysphagia in this population. The earlier diagnosis and intervention was conducted, the less negative effects of oropharyngeal dysphagia happened. As we have mentioned the most common swallowing assessment instrument troubles, taking advantage of other standardized dysphagia evaluation tools such as SDQ seems necessary.

The Cronbach alpha coefficient for original SDQ and the Japanese version of Swallowing Disturbance Questionnaire (SDQ-J) were 0.89 and 0.84, respectively [14, 16]. Similarly, the SDQ-P had a good internal consistency (Cronbach's alpha coefficient = 0.86), so it would be a reliable questionnaire. Then, if a PD patient scores greater than 12 on SDQ-P, he/she should be referred to speech pathologist for a comprehensive swallowing evaluation.

There were no significant differences between patients with or without aspiration in the term of H&Y stage. Generally, we expect more severe dysphagia symptoms and higher PAS scores in advanced stages of the Parkinson's disease; however, it was also claimed that dysphagia and Hoehn & Yahr stage did not always correlate with each other [30]. Therefore, early detection of deglutition problems during the first stages of the disease should be considered in order to decrease the risk of other complications such as aspiration pneumonia.

Finally, it was proved that the results of SDQ could be adversely affected by depression and anxiety, as Manor et al. found the following: “the comparison between patients who scored in the two opposite ends of the anxiety and depression ranges demonstrated that the most anxious and depressed patients reported more swallowing difficulties (SDQ scores) compared with the least anxious and depressed ones” [31]. Thus, dysphagia management team should pay more attention to these factors when they want to interpret the results of this questionnaire.

In summary, our study demonstrates the reliability and also the feasibility of SDQ-P for screening of aspiration in Iranian patients with PD; therefore, administration of SDQ-P in neurology and specifically swallowing clinics is recommended. Evaluation of SDQ-P in a larger subject population and also investigation of SDQ-P for utilizing in other neurological diseases such as multiple sclerosis would be suggested.

## Figures and Tables

**Table 1:** Penetration-Aspiration Scale.

Category	Score	Descriptions
No penetration or aspiration	1	Contrast does not enter the airway

Penetration	2	Contrast enters the airway and remains above vocal folds; no residue
	3	Contrast remains above vocal folds; visible residue remains
	4	Contrast contacts vocal folds; no residue
	5	Contrast contacts vocal folds; visible residue remains

Aspiration	6	Contrast passes glottis; no subglottic residue visible
	7	Contrast passes glottis; visible subglottic residue despite patient's response
	8	Contrast passes glottis; visible subglottic residue; and absent patient response

**Table 2:** Results of the videofluoroscopic swallowing study in patients with Parkinson's disease.

	*N* (M : F)	Mean age	Mean H&Y stage
Aspirated	10 (5 : 5)	68.5 ± 9.9	2.40
Nonaspirated	49 (34 : 15)	65.4 ± 9.7	1.90

Total	59 (39 : 20)	66.0 ± 9.7	1.98

M: male, F: female, and H&Y: Hoehn & Yahr stage.

**Table 3:** Results of the comparison between the Persian version of the questionnaire and videofluoroscopic swallowing study.

SDQ-P score	Normal	Aspiration	Total
≤12	44	0	44
>12	5	10	15
Total	**49**	**10**	**59**
Sensitivity	96.7%		
Specificity	91.2%		
Positive predictive value (PPV)	0.67		
Negative predictive value (NPV)	1.00		
Pretest probability	16.9%		
Posttest probability	66.7%		

SDQ-P: the Persian version of the Swallowing Disturbance Questionnaire.

## Appendix

### Swallowing Disturbance Questionnaire

*Questions*

(1) Do you experience difficulty in chewing solid food like an apple, cookie, or a cracker?
  (Never = 0)
  (Seldom (once a month or less) = 1)
  (Frequently (1–7 times a week) = 2)
  (Very frequently (more than 7 times a week) = 3)
(2) Are there any food residues in your mouth, cheeks, under your tongue or stuck to your palate after swallowing?
  (Never = 0)
  (Seldom (once a month or less) = 1)
  (Frequently (1–7 times a week) = 2)
  (Very frequently (more than 7 times a week) = 3)
(3) Does food or liquid come out of your nose when you eat or drink?
  (Never = 0)
  (Seldom (once a month or less) = 1)
  (Frequently (1–7 times a week) = 2)
  (Very frequently (more than 7 times a week) = 3)
(4) Does chewed up food dribble from your mouth?
  (Never = 0)
  (Seldom (once a month or less) = 1)
  (Frequently (1–7 times a week) = 2)
  (Very frequently (more than 7 times a week) = 3)
(5) Do you feel you have too much saliva in your mouth; do you drool or have difficulty swallowing your saliva?
  (Never = 0)
  (Seldom (once a month or less) = 1)
  (Frequently (1–7 times a week) = 2)
  (Very frequently (more than 7 times a week) = 3)
(6) Do you swallow chewed up food several times before it goes down your throat?
  (Never = 0)
  (Seldom (once a month or less) = 1)
  (Frequently (1–7 times a week) = 2)
  (Very frequently (more than 7 times a week) = 3)
(7) Do you experience difficulty in swallowing solid food (i.e., do apples or crackers get stuck in your throat)?
  (Never = 0)
  (Seldom (once a month or less) = 1)
  (Frequently (1–7 times a week) = 2)
  (Very frequently (more than 7 times a week) = 3)
(8) Do you experience difficulty in swallowing pureed food?
  (Never = 0)
  (Seldom (once a month or less) = 1)
  (Frequently (1–7 times a week) = 2)
  (Very frequently (more than 7 times a week) = 3)
(9) While eating, do you feel as if a lump of food is stuck in your throat?
  (Never = 0)
  (Seldom (once a month or less) = 1)
  (Frequently (1–7 times a week) = 2)
  (Very frequently (more than 7 times a week) = 3)
(10) Do you cough while swallowing liquids?
  (Never = 0)
  (Seldom (once a month or less) = 1)
  (Frequently (1–7 times a week) = 2)
  (Very frequently (more than 7 times a week) = 3)
(11) Do you cough while swallowing solid foods?
  (Never = 0)
  (Seldom (once a month or less) = 1)
  (Frequently (1–7 times a week) = 2)
  (Very frequently (more than 7 times a week) = 3)
(12) Immediately after eating or drinking, do you experience a change in your voice, such as hoarseness or reduced?
  (Never = 0)
  (Seldom (once a month or less) = 1)
  (Frequently (1–7 times a week) = 2)
  (Very frequently (more than 7 times a week) = 3)
(13) Other than during meals, do you experience coughing or difficulty in breathing as a result of saliva entering your windpipe?
  (Never = 0)
  (Seldom (once a month or less) = 1)
  (Frequently (1–7 times a week) = 2)
  (Very frequently (more than 7 times a week) = 3)
(14) Do you experience difficulty in breathing during meals?
  (Never = 0)
  (Seldom (once a month or less) = 1)
  (Frequently (1–7 times a week) = 2)
  (Very frequently (more than 7 times a week) = 3)
(15) Have you suffered from a respiratory infection (pneumonia, bronchitis) during the past year?
  (Yes = 2.5)
  (No = 0.5).

## Abbreviations

PD: Parkinson’s disease
H&Y: Hoehn & Yahr
SDQ: Swallowing Disturbance Questionnaire
SDQ-P: Persian version of the SDQ
SDQ-J: Japanese version of the SDQ
VFSS: Videofluoroscopic swallowing study
FEES: Fiberoptic endoscopic evaluation of swallowing
PAS: Penetration-Aspiration Scale
PPV: Positive predictive value
NPV: Negative predictive value.
